# Transcriptome profile analysis of cell proliferation molecular processes during multicellular trichome formation induced by tomato *Wo*^v^ gene in tobacco

**DOI:** 10.1186/s12864-015-2099-7

**Published:** 2015-10-26

**Authors:** Changxian Yang, Yanna Gao, Shenghua Gao, Gang Yu, Cheng Xiong, Jiang Chang, Hanxia Li, Zhibiao Ye

**Affiliations:** Key Laboratory of Horticultural Plant Biology (Ministry of Education), Huazhong Agricultural University, Wuhan, 430070 Hubei PR China

**Keywords:** Tobacco, Cell proliferation, Transcriptome, Multicellular trichome, *Wo*

## Abstract

**Background:**

Trichomes, developing from the epidermis of nearly all terrestrial plants, provide good structural resistance against insect herbivores and an excellent model for studying the molecular mechanisms underlying cell fate determination. Regulation of trichomes in Rosids has been well characterized. However, little is known about the cell proliferation molecular processes during multicellular trichome formation in Asterids.

**Results:**

In this study, we identified two point mutations in a novel allele (*Wo*^v^) at *Wo* locus. Ectopic expression of *Wo*^v^ in tobacco and potato induces much more trichome formation than wild type. To gain new insights into the underlying mechanisms during the processes of these trichomes formation, we compared the gene expression profiles between *Wo*^v^ transgenic and wild-type tobacco by RNA-seq analysis. A total of 544 co-DEGs were detected between transgenic and wild-type tobacco. Functional assignments of the co-DEGs indicated that 33 reliable pathways are altered in transgenic tobacco plants. The most noticeable pathways are fatty acid metabolism, amino acid biosynthesis and metabolism, and plant hormone signal transduction. Results suggest that these enhanced processes are critical for the cell proliferation during multicellular trichome formation in transgenic plants. In addition, the transcriptional levels of homologues of trichome regulators in Rosids were not significantly changed, whereas homologues of genes (*Wo* and *SlCycB2*) in Asterids were significantly upregulated in *Wo*^v^ transgenic tobacco plants.

**Conclusions:**

This study presents a global picture of the gene expression changes induced by *Wo*^v^- gene in tobacco. And the results provided us new insight into the molecular processes controlling multicellular formation in tobacco. Furthermore, we inferred that trichomes in solanaceous species might share a common network.

**Electronic supplementary material:**

The online version of this article (doi:10.1186/s12864-015-2099-7) contains supplementary material, which is available to authorized users.

## Background

Plant trichomes originate from epidermal cells of nearly all terrestrial plants. Trichomes exist in various shapes and sizes, including unicellular or multicellular, glandular or non-glandular, and branched or unbranched [1]. Trichomes also play important roles in plant adaptation to biotic and abiotic stresses, including improvement of insect resistance, reduction of water loss, and increase of tolerance to extreme temperatures [2, 3]. Among these roles, insect resistance should be improved through spatial hindrance and secretion of toxins. Given that trichomes are easily accessible and their appendages are insignificant for plant growth, they provide an excellent model system for studying the molecular mechanisms in plant cell differentiation [4].

The common network controlling trichome formation in Rosids has been well characterized. However, information about the regulation of trichome initiation in Asterids is very limited. Trichomes in the Rosid *Arabidopsis* typically consist of unicellular structures, and the regulatory pathway of which has been extensively studied. Since the first trichome-related gene, *GLABROUS1* (*GL1*), was cloned, several genes participating in trichome formation have been continuously isolated [5]. The identification of these genes, including *TRANSPARENT TESTA GLABRA1* (*TTG1*), *GLABRA3* (*GL3*), and *ENHANCER OF GLABRA3* (*EGL3*), has elucidated the molecular mechanism of trichome formation [6–8]. These genes induce trichome initiation in the form of a MYB/bHLH/WD repeat complex [9]. This complex can promote the expression of its downstream genes, namely, *GL2*, *TTG2*, *SIAMESE* (*SIM*), and *RETINOBLASTOMA RELATED1* (*RBR1*), thereby inducing trichome formation [10, 11]. Several negative regulators were also characterized, including *CAPRICE* (*CPC*), *TRY*, *ENHANCER OF TRY AND CPC 1* (*ETC1*), *ETC2*, *ETC3*, and *TRICHOMELESS1* (*TCL1*) [12]. Moreover, these negative regulators were activated by the MYB/bHLH/WD repeat complex and thereby inhibit trichome fate [9]. Cell-fate determination during trichome formationmust be controlled by the cell cycle. *CCS52A1* encodes an endoreduplication factor, in which mutation could activate trichome initiation [13]. The ectopic expression of *CYCLIN B1;2* and *CYCD3;1* also separately induces the formation of multicellular trichomes in *Arabidopsis*, implying that more cyclins may be involved in the trichome formation [14, 15]. A Rosid cotton fiber with unicellular trichome-like structure may be controlled by the pathway common with that of the *Arabidopsis* trichome [16]. The ectopic expression of two homologous genes of *GL1*, namely, *GhMYB1* and *GaMYB2*, can restore the trichome phenotype in *gl1* mutant [17, 18]. *GaHOX1* and *GaHOX3* with high sequence similarities to *GL2* could also activate trichome initiation when expressed in *Arabidopsis* [19].

Trichomes in Asterids consist of multicellular structures. The epidermal cells selected as multicellular trichomes will further divide perpendicularly to the epidermal surface. However, whether multicellular trichomes in Asterids share a common molecular mechanism and the event during cell proliferation after trichome cell-fate determination remain unknown. Trichomes in Asterid tobacco (*Nicotiana tabacum*) exhibit multicellular types. The MYB transcription factor *MIXTA* in snapdragon can activate trichome formation when ectopically expressed in tobacco [18]. Overexpression of two other homologues of *MIXTA* (*AmMYBML1* from snapdragon and *CotMYBA* from cotton) in tobacco can also induce multicellular trichome formation [18, 20]. These data demonstrated that several unidentified MIXTA-like genes may participate in the control of multicellular trichome formation in tobacco. Tomato also produces several types of multicellular trichomes. *Woolly* (*Wo*) gene is a HD-ZipIV transcription factor which is responsible for type I trichome formation [21]. The homologue of *Wo* in *Arabidopsis*, i.e., *PROTODERMAL FACTOR2*, participates in the differentiation of shoot epidermal cell but not in trichome initiation [22]. The ectopic expression of a *GL3*-like gene *LC* can also induce trichome formation in *Arabidopsis* but not in tobacco and tomato [23]. Thus, unicellular and multicellular trichomes may be controlled by different regulatory networks. Cell-cycle regulators are also involved in trichome formation. The B-type cyclin gene *SlCycB2* functions in tomato multicellular trichome formation, which shows direct protein–protein interaction with *Wo* [21]. However, whether the interaction between HD-Zip IV regulators and B-type cyclins is conserved in multicellular trichome formation, at least in solanaceous species, remains unclear.

Apparently, cell mitosis is important for plant growth and development. Cell division occurs at specialized meristems. For example, the shoot apical meristem divides and produces new stems and leaves, and the root apical meristem continuously adds new cells to the growing root. Although common basic cell division mechanisms occur between plants and other eukaryotes, plants have evolved some novel characters regulating postembryonic development [24]. Cell division must coordinate with differentiation and development in plants. Therefore, the molecular mechanism of cell proliferation and differentiation is difficult to unfold in plant. Multicellular trichomesserve as important models to characterize the mechanisms of cell proliferation and differentiation. Unfortunately, knowledge on cell proliferation during multicellular trichome formation is limited. Conversely, studies on yeast and cancer cells have facilitated our understanding of the mechanisms of cell proliferation. Cell-cycle progression is controlled by many cyclins and cyclin-dependent kinases . The activities of the complexes between CDKs and cyclins are also monitored by phosphorylation/dephosphorylation [25]. Synthesis of adequate lipids is essential for membrane formation during cell proliferation in cancer cells [26]. Many cancer-related genes also play critical roles in the synthesis and metabolism of lipids and amino acids [27].

Plants must confront the attack of herbivores. Thus, plants have developed several defense systems, including immune response and mechanical protection. Trichomes contribute to plant defense against herbivorous insects and pathogens through physical and chemical deterrents [28]. Previous studies have demonstrated that leaf trichomes can decrease the feeding by herbivores, as well as the damage caused by the feeding [29]. Insect herbivore resistance is positively correlated with trichome density [30]. Studies have shown that trichome-producing *Arabidopsis lyrata* can more effectively protect plants against herbivores than the glabrous types [31]. The number of surviving larvae and adults of leaf miners is negatively related to type I trichome density in tomato [32]. Leafhoppers are effectively captured by high density of hooked trichomes on leaves of field bean cultivars [33]. The growth rate of herbivorous insects is significantly increased when leaf trichomes are removed in *Arabidopsis* [34]. In tomato, methyl jasmonate can negatively affect herbivore populations because of its inducibility to trichome formation [35]. Large amounts of secondary metabolites produced or released by trichomes also provide protection against herbivores, including acyl sugars, methyl ketones, terpenes, and alkaloids [36]. Some proteins in trichomes also exhibit defensive functions, like proteinase inhibitors, polyphenol oxidases, and phylloplanins [37–39].

In this study, we identified a novel allele *Wo*^v^ at the *Wo* locus in woolly mutant LA3560. This allele could induce multicellular trichome formation when ectopically expressed in tobacco. Aphid bioassays also demonstrated that *Wo*^v^ transgenic tobacco plants showed higher resistance levels than those of wild-type (WT) plants. To reveal the underlying mechanisms of cell proliferation during multicellular trichome formation and improvement of aphid resistance, genome-wide analysis of gene transcription was conducted between *Wo*^v^ transgenic tobacco plant and its WT. A total of 714 and 368 genes in transgenic plants were upregulated and downregulated, respectively. The identification of trichome-related genes provided new insights into the molecular mechanisms of multicellular trichome differentiation and subsequent cell proliferation.

## Results

### Identification of novel allele at *Wo* locus

**Fig. 1:**
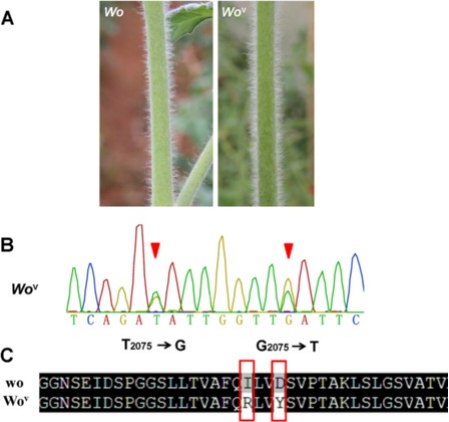
Identification of the mutations in *Wo* ^v^ at *Wo* locus in woolly mutant LA3560. **a** *Wo* ^v^ mutant LA3560 shows much higher trichome density than other alleles; **b** Two nucleotide substitutions takes place in its coding region; **c** Two point mutations result in one amino acid replacement separately

We have identified three alleles (*Wo*^*−*^, *Wo*^*m*^, and *Wo*^*mz*^) at the *Wo* locus from three woolly mutants including LA3186, LA0258, and LA1908 [21]. However, the allele *Wo*^v^ inducing higher trichome density in woolly mutant LA3560 remains uncharacterized (Fig. 1a). We amplified and sequenced the genomic sequences of this locus from LA3560 and determined two nucleotide substitutions in its 3′ portion of the coding region by using *wo* as reference (Accession number: JF518780) (Fig. 1b). Using amino acid analysis, we determined that these two point mutations resulted in one amino acid replacement (Ile-692 to Arg, Asp-695 to Tyr) separately (Fig. 1c). In addition, the full-length cDNA of *Wo*^v^ contains a 2,193 bp open reading frame, encoding a polypeptide of 730 amino acids (Additional file 1).

### Ectopic expression of tomato *Wo*^v^ in tobacco and potato induces multicellular trichome formation

**Fig. 2:**
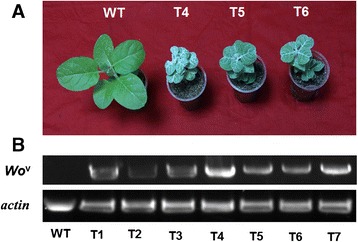
Trichome phenotype (**a**) and *Wo* ^v^ expression in the transgenic tobacco plants (**b**)

**Fig. 3:**
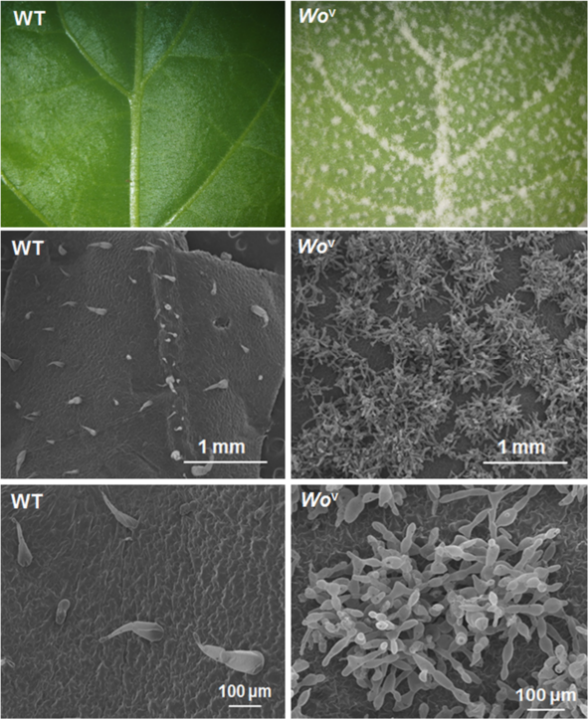
SEM observation of leaf trichomes in the transgenic tobacco plants

To investigate whether the formation of trichomes in tobacco and tomato is controlled by the common network, we generated a construct by placing the open reading frame of *Wo*^v^ under the control of its native promoter. We introduced this construct into *N. tabacum* by *Agrobacterium*-mediated transformation. We then obtained nine individual kanamycin-resistant tobacco transgenic lines (T_0_). These transgenic plants were further examined by PCR using genomic DNA as template and gene-specific primers (Additional file 2). Finally, seven positive transformants were harvested. The *Wo*^v^ transgenic tobacco plants were densely covered with trichomes (Fig. 2a). The transcript levels of the *Wo*^v^ gene in young leaves and WT plants were analyzed by semi-quantitative RT-PCR. The expression level of *Wo*^v^ gene in transgenic plants was significantly upregulated, whereas no expression of target gene existed in the WT plants (Fig. 2b). The trichome phenotype was further observed under the stereomicroscope, which displays snowflake-like images (Fig. 3). Previous studies demonstrated that tobacco produces short- and long-stalked multicellular trichomes. To investigate which trichome types result in the phenotypic changes, we observed the trichome by SEM. A new type characterized by numerous cells in one trichome without glands replaces the two types of trichomes mentioned above (Fig. 3). Simultaneously, the *Wo*^v^ ectopic expression in tobacco causes the formation of malformed flower with shorter style and shorter petal than those of WT (Additional file 3). Notably, *Wo*^v^ can also induce trichome formation when expressed in potato (Additional file 4).

### Aphid bioassays

**Fig. 4:**
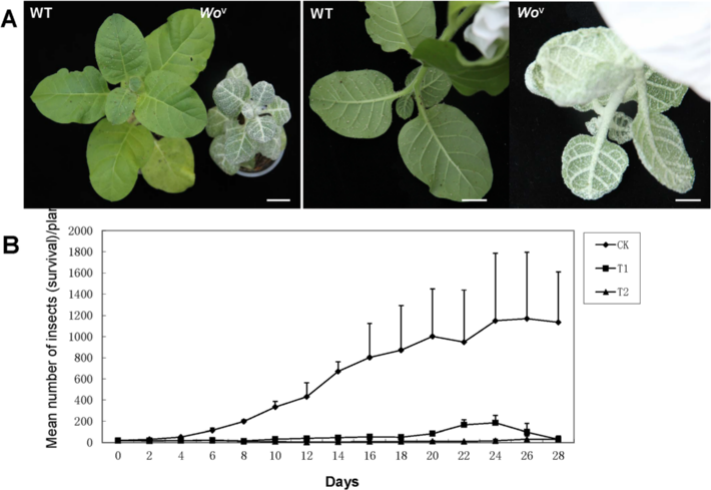
Phenotype of aphid resistance (**a**) and aphid bioassay on transgenic tobacco plants (**b**). WT: wild type; T1 and T2: two transgenic tobacco plants. (Scale bars: 1.0 cm.)

Three clonal replicated plants of two independent transgenic tobacco T_0_ lines and untransformed tobacco plants were used to perform bioassays with late instar aphid nymphs. Investigation of the aphid survival and growth demonstrated that transgenic tobacco plants with multicellular trichomes exhibited significantly lower levels of resistance against aphids and lower growth of the aphid population than those of the untransformed controls (Fig. 4a). The number of aphids in transgenic tobacco plants did not significantly increase, whereas that in the controls rapidly increased after 6 d bioassay (Fig. 4b). The mean of aphids on transformants was significantly lower throughout the assay period than that of the controls (*p* < 0.05). The aphid number reached the highest point at 24 d after inoculation (from 20 aphids per plant to 1870 aphids per plant). These data indicated that aphid resistance was closely related to trichome phenotype.

### Enhancement of photosynthetic activity

The photosynthetic rate in transgenic tobacco was nearly four times more than that in WT. The stomatal conductance and transpiration rate in transgenic tobacco were nearly three times more than those in WT. The results of photosynthetic activity demonstrated that the trichome phenotype significantly decreased photosynthetic rate, stomatal conductance, and transpiration rate in the *Wo*^v^ transgenic tobacco plants compared with the untransformed plants (Additional file 5). We concluded that photosynthesis, which plays an important role in biological processes, was significantly depressed in the T plants based on these physiological data.

### Global analysis of RNA-seq data

The molecular mechanism regulating unicellular trichome formation in *Arabidopsis* is well characterized. However, multicellular trichome, which may be controlled by a distinct pathway, is not deeply studied. As *Wo*^v^ gene can induce the formation of multicellular trichomes consisting of dozens of cells when ectopically expressed in tobacco plants, we reasoned that analysis of transcript profiles between *Wo*^v^ transgenic (T) and WT plants would reveal the underlying mechanisms of multicellular trichome formation. To identify gene expression changes that led to multicellular trichome formation, four mRNA samples were collected from these materials, two representing transgenic and two representing WT plants. About 20 million raw reads were produced for each sample (Table 1). Clean reads were also obtained by discarding low-quality reads, resulting in 14914241 (85.87 %) clean reads for T1, 16931963 (84.53 %) clean reads for T2, 18039905 (85.93 %) clean reads for WT1, and 17073339 (86.32 %) clean reads for WT2. The sequence length of the vast majority of all clean reads was between 86 and 87 bp. To assess the quality of the RNA-seq data, the reads were mapped to the *N. tabacum* reference genome. Among them, 12898573 (86.48 %) clean reads for T1, 15087130 (89.1 %) clean reads for T2, 16199408 (89.8 %) clean reads for WT1, and 15361793 (89.98 %) clean reads for WT2 were successfully mapped to the reference genome (Table 1). The percentage of the unique mapping reads is approximate 75 % in each sample, suggesting that the RNA-seq libraries exhibited high quality. Correlation efficient of the gene expression between two biological replicates indicated high repeatability (R_T_^2^ = 0.884, R_WT_^2^ = 0.93) (Additional file 6). These sequencing assessments demonstrated that the RNA-seq data were sufficient for subsequent gene expression analyses.

**Table 1:** Summary statistics of the high-quality RNA-seq reads number in transgenic tobacco plants (T) and its wild type (WT)

	T1	T2	WT1	WT2
Total mapped	12898573 (86.48 %)	15087130 (89.1 %)	16199408 (89.8 %)	15361793 (89.98 %)
Unique mapped	11018603 (73.88 %)	12842110 (75.85 %)	13739693 (76.16 %)	13049564 (76.43 %)
Multiple mapped	1879970 (12.61 %)	2245020 (13.26 %)	2459715 (13.63 %)	2312229 (13.54 %)
Raw reads	17367232	20029812	20992196	19779274
Clean reads	14914241 (85.87 %)	16931963 (84.53 %)	18039905 (85.93 %)	17073339 (86.32 %)
Reads > 0	22441(91.85 %)	22566(92.36 %)	22585(92.40 %)	22530(92.22 %)
Reads > 10	17178(70.31 %)	17805(72.88 %)	17515(71.69 %)	17432(71.35 %)

### Functional annotation and differential expression of genes between T and WT plants

**Fig. 5:**
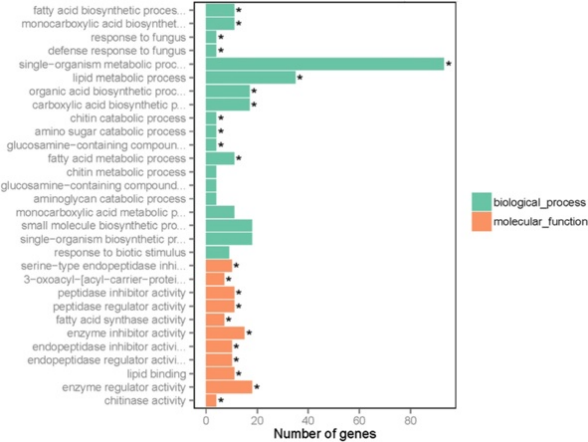
Functional categorization of the up-regulated genes between the *Wo* ^v^ transgenic tobacco plants and the wild-type. *represents the significant enrichment GOterms

Differentially expressed genes were identified using DESeq (version 1.10.1) with an adjusted *p*-value <0.05. Genes with FPKMs in the interval 0–1 were considered to be expressed at a low level, and genes with FPKMs over 60 were present at a very high level. In addition, the distributions of the gene expression values showen no obvious differences for the four samples (Table 2 and Additional file 6). In this study, a total of 381 and 163 genes were upregulated and downregulated in transgenic plants, respectively (Additional file 7). Obviously, there were no too many genes activated or inhibited by exogenous *Wo*^v^ gene. This suggested these DEGs may be closely related to the target phenotype. In addition, differences of the up-regulated genes ranged from1.9-fold to 9.1- fold, and differences of the down-regulated genes ranged from 1.8-fold and 9.3- fold. To evaluate the functional categories of the genes with significant transcriptional changes between T and WT, we analyzed these DEGs by using the GOseq based Wallenius non-central hyper-geometric distribution. The categorization of DEGs according to biological processes, molecular functions, and cellular components was analyzed. Categories based on biological processes demonstrated that the upregulated genes were mainly involved in fatty acid biosynthetic process, monocarboxylic acid biosynthesis, response to fungus, and lipid metabolic process (Fig. 5). By contrast, the downregulated genes were mainly related to phosphorelay signal transductiion, inositol catabolic process, alcohol catabolic process, and polyol catabolic process (Additional file 8). Based on molecular function, the upregulated genes in T were finally classified into 11 categories, including serine-type endopeptidase inhibitor activity, 3-oxoacyl-[acyl-carrier-protein] reductase, peptidase inhibitor activity, peptidase regulator activity, and fatty acid synthase activity (Fig. 5). Aside from phosphorelay response regulator, the down-regulated genes were mainly enriched in the categories of terpene synthase activity, carbon − oxygen lyase activity, and iron ion binding (Additional file 8). When we classified the DEGs according to cellular components, we found that there was no overrepresented GO terms for upregulated genes, whereas those for the downregulated genes were external encapsulating structure, cell wall, and apoplast (Fig. 5 and Additional file 8).

**Table 2:** Distribution of gene expressions in the T and WT tobacco plants

FPKM Interval	T1	T2	WT1	WT2
0 ~ 1	19400(38.26 %)	19545(38.55 %)	19952(39.35 %)	20242(39.92 %)
1 ~ 3	10232(20.18 %)	9665(19.06 %)	10137(19.99 %)	9843(19.41 %)
3 ~ 15	14117(27.84 %)	14268(28.14 %)	13666(26.95 %)	13679(26.98 %)
15 ~ 60	5406(10.66 %)	5498(10.84 %)	5182(10.22 %)	5229(10.31 %)
>60	1550(3.06 %)	1729(3.41 %)	1768(3.49 %)	1712(3.38 %)

### Identification of known trichome-related genes

Trichomes originate from the aerial epidermis of nearly all land plants, existing in unicellular and multicellular forms. Previous studies indicated that these two types of trichomes may be controlled by different pathways. In recent years, many key regulators and their downstream genes participating in the regulation of trichome formation have been identified. For example, *GL1*, *TTG1*, and *GL3* in *Arabidopsis*; *GhMYB1* and *GaMYB2* in cotton; *PtMYB186* in poplar; and *Wo* and *SlCycB2* in tomato have been identified. Detailed information about these genes is listed in Additional file 9 [5–7, 17, 40]. The ectopic expression of *Wo*^v^ in *N. tabacum* induces the formation of multicellular trichomes on the entire aerial epidermis, indicating that the unidentified *Wo*-like protein and the corresponding regulatory network control trichome differentiation in tobacco. To identify transcriptional changes of trichome-related genes in the current study, we performed alignments of these known genes with all the detected unigenes in tobacco. The expression levels of homologues of genes regulating trichome formation in Rosids (*Arabidopsis*, cotton and poplar) did not change significantly, whereas those in Asterids were significantly upregulated (tomato) (Additional file 9). Notably, the expression of gene_2092 (homolog of *Wo* gene), gene_49227 and gene_22166 (homologs of *SlCycB2* gene), and gene_22022 and gene_37943 (homologs of *PDF1* gene, without function in Rosids trichome formation) increased significantly in the *Wo*^v^ transgenic tobacco plants (Additional file 9). Therefore, we inferred that tomato and tobacco share the common pathway of trichome formation. More than one gene also participates in the regulation of trichome initiation in tobacco. As we know, transcription factors (TFs) play important roles in regulating the expression of their downstream genes. Putative TF genes were aligned with the known TFs. In the current study, we identified 51 putative TFs belonging to 15 TF families during the DEGs between T and WT plants (Additional file 10). The ARR family was the most prominent, followed by the HD-ZIP family. Notably, the majority of these families were significantly upregulated in T plants, indicating that ethylene signaling pathway and MYB TFs may induce multicellular trichome initiation and subsequent cell proliferation.

### Identification of putative pathways controlling multicellular trichome formation

**Fig. 6:**
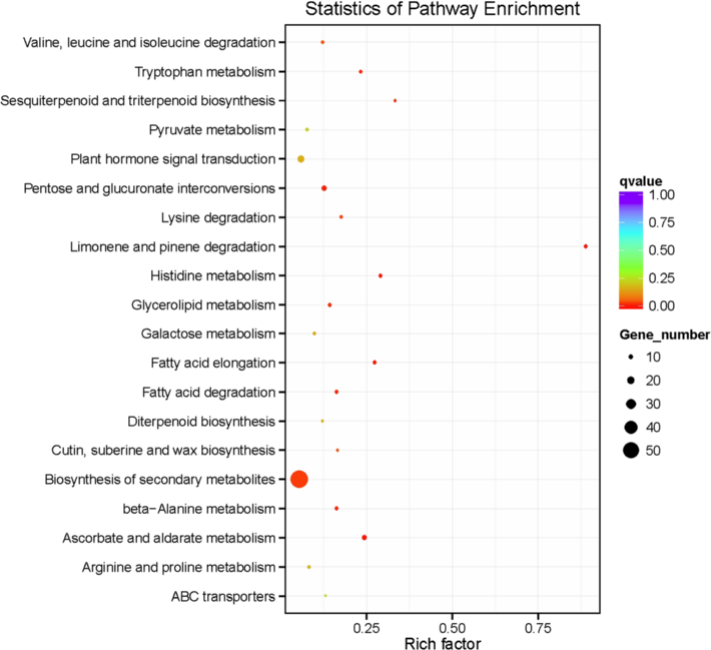
Scatterplot of enriched KEGG pathways for differentially expressed genes between T and WT

*Wo*^v^ transgenic tobacco plants induce the formation of multicellular trichomes, which consist of many cells. For biological interpretation of the differential expression genes, we aligned these genes with the reference canonical pathways in KEGG using KOBAS 2.0. In this study, 398 out of 544 DEGs were mapped to 74 KEGG pathways. These pathways are shown in Additional file 11. Moreover, a total of 33 different pathways were identified with more than 3 genes affiliated, some of which were consistent with biological processes characterized by GO analyses. We found that the biosynthesis pathway of secondary metabolites was significantly enriched for the DEGs between T and WT (Fig. 6). It may result from the disappearance of glandular trichomes which were replaced by the non-glandular trichomes in *Wo*^v^ transgenic tobacco plants. In addition, some of these pathways might be correlated with the formation of target traits, including fatty acid metabolism, amino acid biosynthesis and metabolism, and plant hormone signal transduction [26, 41, 42].

Phytohormones, including gibberellin (GA), jasmonic acid (JA), and ethylene (ET), play important roles in *Arabidopsis* trichome initiation and branching [12]. However, little is known about their functions in multicellular trichome formation. Notably, we found several genes participating in plant hormone signal transduction. For example, homologs for the genes (EIN1, RAP27, DRE2A, AHK4, etc.) in the ET pathway were identified (Additional file 10). Furthermore, ARR3, 4, 6, 8, and 9 functioning downstream of ethylene signal transduction as signalling components were also characterized (Additional file 10). Simultaneously, several homolog genes involved in auxin signal pathway, such as indole-3-acetic acid-amido synthetase GH3.5, AUX22, and SAUR14, were also detected. However, genes participating in JA and GA signal transduction pathways were identified in 544 DEGs, which were not insignificantly changed. Thus, ET and IAA are involved in multicellular trichome initiation and subsequent cell proliferation in multicellular trichomes, whereas JA and GA may not.

### Verification of *Wo*^v^-related genes

**Fig. 7:**
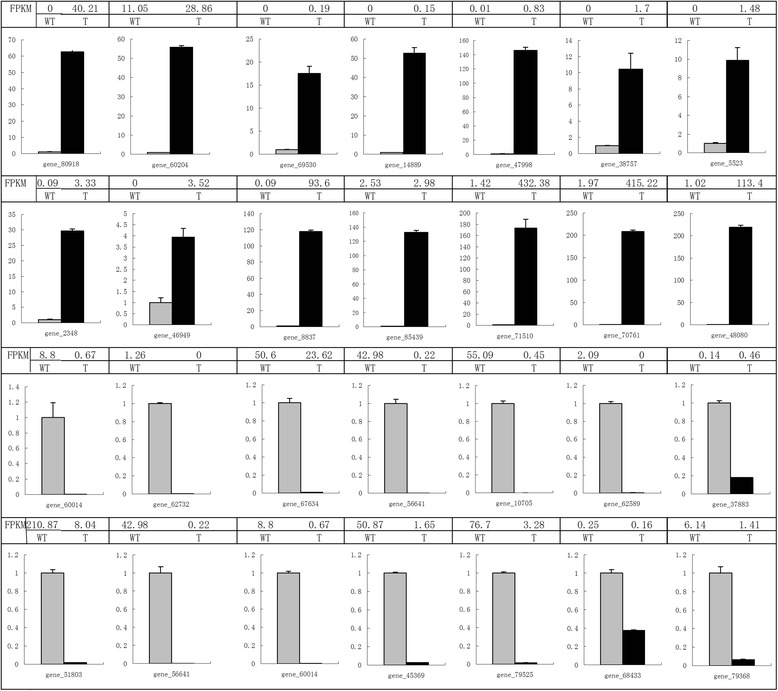
Real-time Q-RT-PCR confirmation of the differentially expressed genes between the wild type (grey columns) and the *Wo* ^v^ transgenic tobacco plants (black columns). Columns and bars represent the means and standard error (*n* = 3) respectively. The transcript abundance from RNA-seq data was displayed on the top of each gene

**Fig. 8:**
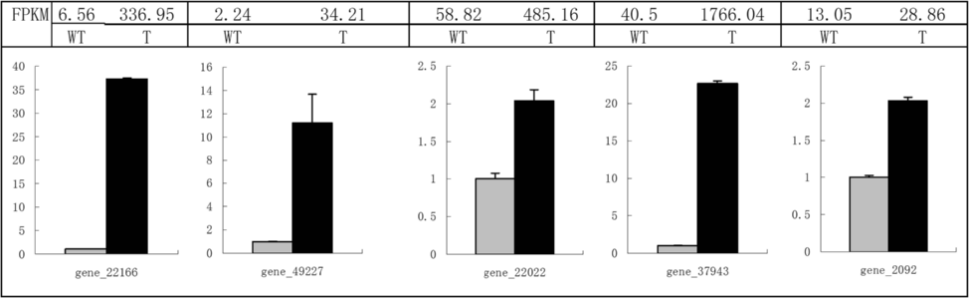
Real-time Q-RT-PCR confirmation of the homologues of *Wo* and *SlCycB2* between the *Wo* ^v^ transgenic tobacco plants and the wild-type. Columns and bars represent the means and standard error (*n* = 3) respectively. The transcript abundance from RNA-seq data was displayed on the top of each gene. RPKM, reads per kilo base of a gene per million reads

To confirm the RNA-seq results, quantitative RT-PCR was conducted on the randomly selected *Wo*^v^-related genes based on the RNA-seq data. Transcriptional change revealed by RNA-seq was also validated in independent experiments. A total of 28 significantly differentially expressed genes, including 14 up-regulated genes and 14 down-regulated genes, were selected to design the gene-specific primers (Additional file 2). The patterns of transcript abundance between WT and T were compared with RNA-seq data. Although some quantitative differences exist at expression level, qRT-PCR results indicated that all of the 28 genes displayed the similar expression tendency as the RNA-seq data. Figure 6 shows the expression levels of the 28 genes between MT and T. For instance, the transcript level of germin-like gene_80918 was up-regulated 62.6 times more in MT than in T as analyzed by quantitative RT-PCR, which was consistent with RNA-seq data demonstrating that the gene expression in the T was 8.9-fold higher than that in the WT. The expression level of the rest of the genes, including defensin-like gene, osmotin 34, serine protease inhibitor, RING/U-box gene, SAUR-like auxin-responsive gene, and six other newly detected genes with significant transcriptional changes without homologues in *Arabidopsis* (gene_60204, gene_69530, gene_47998, gene_46949, gene_8837, and gene_48080), were analyzed between the WT and transgenic plants (Fig. 7). As expected, the transcription levels of defensin-like gene, osmotin 34, and serine protease inhibitor were significantly upregulated in T versus WT, whereas those of RING/U-box and SAUR-like auxin-responsive genes were significantly downregulated in T compared with WT. These findings correlate well with the RNA-seq data. Notably, the expression level of the three homologs of tomato trichome-related genes (gene_2092, gene_49227, and gene_22166) in T is higher than that in WT, which showed a very good correlation with the RNA-seq results (Fig. 8).

## Discussion

### More trichome formation in *Wo*^v^ mutant may depend on changes in stability and activity of allele resulting from two point mutations

The *Wo* gene encodes the HD-ZIPIV protein, which contains four conserved domains (HD domain, bZIP motif, START domain, and SAD domain). Each of the three alleles at *Wo* locus contains an amino acid substitution in the conserved domains [21]. We identified that two point mutations in *Wo*^v^ resulted in replacement of two amino acids. Notably, one of the polymorphic sites in Wo^v^ is also highly conserved in its homologues from various organisms. Therefore, these two point mutations may cause much drastic changes in the stability and activity in Wo^v^ and induce more trichome formation.

### Multicellular trichomes in solanaceous species may share common regulatory pathway

The ectopic expression of unicellular trichome-related gene *GL1* in tobacco failed to induce trichome formation [18]. *MIXTA* could not restore the *gl1* mutant trichome phenotype when expressed in *Arabidopsis*, although it participated in multicellular trichome formation in tobacco [18]. Therefore, the formation of multicellular trichomes may be controlled by a distinct pathway from unicellular trichomes. However, whether trichomes in solanaceous species share the common network remains unknown. In the current study, we identified that ectopic expression of tomato woolly phenotype controlled gene *Wo* in tobacco could lead to multicellular trichome formation. The trichomes were also induced when this gene was introduced into another solanaceous plant, such as potato. Moreover, the expression of homologs of *Wo* (gene_2092) and *SlCycB2* (gene_49227 and gene_22166) was significantly upregulated in the *Wo*^v^ transgenic tobacco plants, whereas that in unicellular trichome formation was unchanged. Therefore, multicellular trichomes in solanaceous species may share the common regulatory pathway mediated by HD-ZIPIV and SlCycB2 proteins.

### Identification of regulatory pathways involved in cell proliferation during multicellular trichome formation in *Wo*^v^ transgenic tobacco

Tobacco produces short- and long-stalked multicellular trichomes, which may be controlled by different regulatory networks [43]. However, genes participating in trichome formation largely remain unknown. Multicellular trichomes were induced when the *Wo*^v^ gene was introduced into tobacco plants. Each of this new type of trichomes contains at least a dozen of cells because of quick cell proliferation after trichome initiation. To reveal the underlying mechanisms of multicellular trichome induction and cell proliferation, we analyzed the transcript expression profiling in *Wo*^v^ transgenic tobacco plants and controls. To avoid variation for all samples, we used two independent transgenic tobacco plants as biological replicates. High correlation efficient of the raw data between T and WT demonstrated that the data obtained from RNA-seq were repeatable and reliable. We found 1082 differentially expressed genes that were changed at least twofold in the T.

Cell division and cell growth depend on nutrients. Thus, the regulation of the availability of nutrients must function in cell proliferation [27]. Previous studies showed that lipid and fatty acid synthesis and metabolism are required for cell proliferation. Thus, enzymes involved in these processes also play important roles. However, whether fatty acid synthesis and metabolism participate in cell proliferation in plant trichome formation remains unknown. In the current study, many genes that were correlated with these processes were identified. Functional cluster analyses demonstrated that many important pathways may participate in the cell proliferation in multicellular trichome formation. The noticeable pathway is the fatty acid synthesis and metabolism. Twenty differentially expressed genes were involved in the fatty acid synthesis and metabolism. These genes included lipoxygenase (encoded by gene_55786), 3-hydroxyacyl-CoA dehydrogenase (encoded by gene_32976), acyl-CoA oxidase 4 (encoded by gene_22033), 3-ketoacyl-CoA synthase 5 (encoded by gene_3684), 3-ketoacyl-CoA synthase 10 (encoded by gene_19674), 3-ketoacyl-CoA synthase 11 (encoded by gene_67695), and 3-ketoacyl-CoA synthase 20 (encoded by gene_26163). The transcription levels of these genes were significantly upregulated in the *Wo*^v^ transgenic tobacco plants. These enzymes play important roles in fatty acid synthesis and metabolism. Saturated very-long-chain fatty acids serve function in cotton fiber formation [44]. Some amino acids were also reported as critical regulators of several cell signaling pathways in cell proliferation of cancer [45] gene_55049, gene_7239, gene_21618, gene_30065, gene_40340, gene_20744, and gene_48928 detected by RNA-seq in T were at least two times higher than those in WT, which encodes 4-hydroxyphenylpyruvate dioxygenase, methylcrotonyl-CoA carboxylase, arginine decarboxylase 2, glutamine-dependent asparagine synthase 1, methylenetetrahydrofolate reductase, methionine gamma-lyase, and lysine-ketoglutarate reductase, respectively. These enzymes play pivotal roles in the catalytic action of amino acid metabolisms. Therefore, fatty acids and amino acid-related genes could induce cell proliferation of multicellular trichome formation. Consistent with our results, fatty acid biosynthesis pathways were significantly upregulated in multicellular trichomes of *Artemisia annua* [46].

### Transcriptional regulation of genes correlated with phytohormones in T versus WT

Plant growth and development are extensively modulated by phytohormones. These phytohormones may also function in controlling unicellular trichome formation in *Arabidopsis*. For example, exogenous GA can induce trichome initiation in the glabrous GA-deficient mutant *gal-3* [47]. Application of CK causes trichome formation on the inflorescence stem [48]. JA also functions as an important signaling factor in *Arabidopsis* trichome formation [49]. Many genes related to signaling pathway of phytohormones were also changed in the current study. ET plays diverse roles in plant growth, developmental processes, and stress responses. Notably, the signal transduction of ET was affected in the transgenic tobacco plants. Several genes involved in the ET signal transduction pathway were upregulated. EIN1 belongs to the ET receptor family. RAP27 and AHK4 function as ET-responsive factors [50]. Furthermore, ARR3, 4, 6, 8, and 9 are known as downstream factors of ethylene signal transduction [51]. The ET signaling pathway participates in the control of trichome development and its branching. When our results based on transgenic tobacco plants with multicellular trichomes were compared with those on other plants with trichome phenotypes, ET may have conserved functions in the formation of different trichome types. For example, the transcript abundance of many ET response genes and an ET receptor ETHYLENE RESPONSE SENSOR 1 were significantly changed in polar *fuzzy* mutant [41]. The ET-responsive element-binding factors were highly expressed in mature *Arabidopsis* trichomes [51]. Similarly, ET biosynthesis is one of the most significantly upregulated biochemical pathways during fiber elongation [52]. Multicellular trichomes in *Wo*^v^ transgenic tobacco plants also exhibit many branches. All these findings suggest that ET plays an important role in the formation of multicellular trichomes. To date, excessive reports exist on auxin-regulating trichome formation. In a recent study in tomato, SlIAA15-RNAi transgenic plants demonstrated that auxin is also required for multicellular trichome initiation [53]. Auxin could promote the formation of protocorm trichomes in *Spathoglottis plicata* [54]. Through comparing transcription between transgenic and WT tobacco, apparent upregulation of indole-3-acetic acid-amido synthetase GH3.5, AUX22, and SAUR14 involved in auxin signal transduction pathway were also detected. GA induces trichome initiation by upregulating the transcription level of *GL1* in *Arabidopsis. GLABROUS INFLORESCENCE STEMS* (*GIS*) regulates trichome branching through GA signaling in *Arabidopsis* [42]. Application of GA promotes fiber elongation, indicating that GA also plays an important role in regulation of fiber elongation [55]. However, we did not identify any gene involved in the GA pathway that was significantly upregulated in transgenic tobacco. Therefore, ET and IAA participate in multicellular trichome formation and subsequent cell proliferation in multicellular trichomes.

### Transgenic tobacco plants with multicellular trichomes showed good aphid resistance and lower photosynthesis activity

Plant trichomes could reduce herbivore attack by physical and/or chemical resistance [56]. Thus, we investigated the number of aphid survival in transgenic tobacco plants with multicellular trichomes and found that transgenic tobacco plants showed much higher aphid resistance than WT. Leaf trichome density is positively related to herbivore resistance [3]. Therefore, high leaf trichome density of *Wo*^v^ transgenic tobacco plants could impede the feeding and movement of aphids, thereby increasing the aphid resistance. Cutin and wax could provide protection from insect herbivores [57]. The increase of cutin and wax may also mediate responses to herbivores. Many JA responsive genes are significantly upregulated in transgenic tobacco plants. Therefore, JA pathway may also contribute to the aphid resistance in tobacco. Moreover, many defense-responsive factors were activated in transgenic tobacco plants, which may also improve the aphid resistance. Notably, photosynthetic activity was significantly declined in the transgenic tobacco plants. Based on the RNA-seq results, the transcription of several genes participating in chloroplast formation was significantly repressed in transgenic tobacco plants. Sufficient chloroplasts existed because of quick cell proliferation in multicellular trichome. Therefore, photosynthetic activity is insufficient in transgenic tobacco plants.

## Conclusion

In summary, our results provide global gene expression profiles in tobacco induced by the tomato *Wo*^v^ gene. Analysis of the transcriptional data shows that multicellular trichomes in Asterids (at least in solanaceous species) share a common regulatory network. *Wo*^v^ may induce multicellular trichome formation in tobacco by triggering biosynthesis and metabolism of fatty and amino acids, which are major requirements for cell proliferation. Similar to unicellular trichomes, phytohormones also participate in multicellular trichome formation and subsequent cell proliferation in multicellular trichomes, including ET and IAA. Transgenic tobacco plants with multicellular trichomes showed better aphid resistance than WT. On the one hand, high leaf trichome density could impede the feeding and movement of aphids. On the other hand, the expression of many defense genes was induced by *Wo*^v^ gene.

## Methods

### Plant materials

*Wo*^v^ transgenic tobacco plants and their WT species (*N. tabacum*) were grown in a naturally illuminated glasshouse in the experimental field of the Huazhong Agricultural University. Environmental control (pest and water control) was implemented based on standard protocols [58].

### Identification of *Wo*^v^ at *Wo* locus

Tomato woolly mutant LA3560 was supplied by the Tomato Genetics Resource Center. We amplified and sequenced the genomic and cDNA sequences of *Wo*^v^ at *Wo* locus with the primers described by Yang et al. [21]. Alignment of these sequences was performed with ClustalW (http://www.ebi.ac.uk/Tools/msa/clustalw2/) and edited with GeneDoc 2.7 by using the *wo* sequence as reference.

### Isolation of *Wo*^v^, construction of overexpression vector, and tobacco and potato transformation

We amplified a 3.5 kb promoter fragment upstream the transcription start site from LA3560 genomic DNA and full-length cDNA coding sequence of *Wo*^v^ with the primers listed in Additional file 2. Both the reverse primer of the promoter and the forward primer of the coding region contain the common restriction enzyme recognition site. These two fragments were ligated and inserted into the binary vector pMV2 [21]. The construct *P*_*Wo*_*::Wo* was introduced into the *Agrobacterium* strain C58. We transformed the construct into *N. tabacum* using the method described by Horsch et al. [59]. Transgenic potato plants were obtained according to the method described by Si et al. [60]. Transgenic tobacco and potato plants were identified using genomic DNA as template and gene-specific primers.

### Scanning electron microscopy

Trichomes on fully expanded leaves of transgenic tobacco plants were initially observed under a stereomicroscope. The type of trichomes was identified by scanning electron microscopy (SEM). SEM procedure was performed as follows. Leaves and stems were fixed in 2 % glutaraldehyde and dehydrated in a graded ethanol series. The samples were then sputter coated with gold and analyzed under a JSM-6390/LV scanning electron microscope.

### Aphid bioassay

Tobacco aphids (*Myzus nicotianae*) were challenged by two independent transgenic tobacco T_0_ lines (three clonal replicate plants per line) and untransformed tobacco controls. Tobacco aphids were maintained on mature tobacco plants in a controlled environment growth chamber (24 ± 2 °C, 70 % relative humidity, and 16:8 h light/dark photoperiod). Each plant was confined to a cage assembled with insect proof net, and 20 nymphs of aphids were inoculated on tobacco leaves. The growth rate of the aphid population was calculated each day for an 28 d period after inoculation.

### Photosynthesis activity measurement

Measurements of photosynthesis rate, stomatal conductance, and transpiration rate were performed with a gas exchange system (Li-6400, Li-COR). Three clonal replicate plants per transgenic line and negative transformed line were measured between 11:00 and 12:30 on sunny days. Descriptive statistics and *t*-test were conducted using SPSS 10.0 software. Difference of compared data was considered significant at 1 % level (*p* < 0.05).

### RNA preparation

Two independent *Wo*^v^ transgenic tobacco plants with multicellular trichomes and two negative regenerated plants were used for transcriptomic analysis. Young leaf samples were harvested from three plants, which were generated from the two transgenic plants and one control plant. All samples were immediately frozen in liquid nitrogen, and total RNA was isolated using TRIzol reagent (Invitrogen). The integrity of all 12 RNA samples was examined by 1.2 % agarose gel electrophoresis after treatment with RQ1 DNase (Promega). The quality and quantity of these RNA samples were further determined by measuring the absorbance at 260/280 and 260/230 nm by using SmartSpec Plus spectrophotometer (Bio-Rad).

### RNA-seq and data processing

The materials used for RNA-seq analyses were three young primary leaves from the transgenic and control plants. Up to 10 μg of total RNA was sent to ABlife Inc. (Wuhan, China) where the libraries were produced. The cDNA libraries were then sequenced in BGI Inc. (Shenzhen, China) by using Illumina HiSeq™ 2000 by 100 nt pair-end sequencing. RNA-seq was performed as previously described [61]. Briefly, fragmentations of the purified mRNA were treated by end repair and 5′ adaptor ligation. Reverse transcription was then completed with RT primer to harbor 3' adaptor and randomized hexamer. The obtained cDNA fragments were purified and amplified. The directional RNA-seq libraries were constructed using 200 bp to 500 bp PCR products. When these cDNA libraries were sequenced, clean reads were obtained by removing the adaptors, low quality reads (the proportion of the low quality bases of quality value ≤5 exceed 50 % in one read), and any reads containing unknown bases (>10 %), and the redundant reads, And the clean reads were aligned to the *N. tabacum* genome (ftp://anonymous@ftp.solgenomics.net/genomes/Nicotiana_tabacum) using TopHat (2.0.12) software [62]. Based on the length of the gene and reads count uniquely mapped to this gene, gene expression levels were calculated using FPKM method. This method eliminated the discrepancy of gene expression resulted from varied gene length and sequencing. Therefore, the data of gene expression could be directly used to compare the gene expression differences among target samples.

### Functional analysis of differentially expressed genes

We used the DESeq software to identify the differentially expressed genes between samples, which were specific for differential expression analysis of the RNA-Seq data with biological replicates [63]. An adjusted *P*-value <0.05 found by DESeq are applied as standards to characterize the significance of gene expression level. To characterize the putative functions of differentially expressed genes, Gene Ontology (GO) term analysis was also performed using GOseq based Wallenius non-central hyper-geometric distribution [64] (Young et al., 2010). For further identification of pathways significantly influenced by the *Wo*^v^ gene, KEGG enrichment pathways analysis of DEGs was implemented by the KOBAS (2.0) software by a hypergeometric test and the Benjamini-Hochberg FDR correction (FDR ≤ 0.05) [65].

### Real-time quantitative RT-PCR analysis

Genes were selected for verification by real-time quantitative RT-PCR. Primers were designed using the Primer Premier 5 [66]. Product length was between 80 and 200 bp. All primers used in this analysis are listed in Additional file 2. Total RNA was isolated with TRIzol reagent (Invitrogen). First-strand cDNA was synthesized using PrimeScript reverse transcriptase (TaKaRa). Real-time quantitative PCR was performed in the Roche LightCycler 480 system. Specifically, the reaction mixture contains 2 μl of primers (2.5 μM), 10 μl of SYBR Green I Master Mix (Roche), 2 μl of cDNA template, and 6 μl of water. The real-time PCR program was performed as previously described and with three replicates for each sample; data were indicated as means ± SD (*n* = 3) [21].

### Availability of supporting data

The tobacco transcriptome data are available at http://www.ncbi.nlm.nih.gov/geo/query/acc.cgi?acc=GSE72310.

## Additional files

Additional file 1:
**The sequence information of** ***wo*** **gene and its allele** ***Wo*** ^**v**^ **gene.** (DOCX 17 kb) Additional file 2:
**The primers used for the confirmation of RNA-seq data and positive detection of transgenic tobacco plants.** (XLS 28 kb) Additional file 3:
***Wo*** ^***v***^ **ectopic expression in tobacco plants (T4, T5 and T2) causes the formation of malformed flower, such as shorter style and shorter petal than wild type (WT).** (TIFF 104 kb) Additional file 4:
**Trichome phenotype of** ***Wo*** ^**v**^ **ectopically expressed potato (** ***Wo*** ^**v**^ **) and wild type plants (WT).** (TIFF 278 kb) Additional file 5:
**Comparison of the rate of photosynthesis, stomatal conductance and transpiration rate between** ***Wo*** ^**v**^ **transgenic tobacco plants (T1 and T2) and wild type (WT).** (TIFF 12 kb) Additional file 6:
**Analysis of the gene expression correlation between the two indicated pairs of samples (T1 versus WT1, T2 versus WT2) (A), and boxplot of the log transformed FPKM expression values across four samples (B).** (TIFF 100 kb) Additional file 7:
**The list of all differentially expressed genes between T and WT.** (XLS 103 kb) Additional file 8:
**Functional categorization of the down-regulated genes between the** ***Wo*** ^**v**^ **transgenic tobacco plants and the wild-type.** (JPEG 203 kb) Additional file 9:
**Expression patterns of the homologues of the known trichome-related genes in transgenic tobacco plants.** (XLS 28 kb) Additional file 10:
**Differentially expressed transcription factors found in the** ***Wo*** ^**v**^ **transgenic tobacco plants compared with its wild type.** (XLS 34 kb) Additional file 11:
**The list of all KEGG pathways of the differentially expressed genes.** (XLSX 15 kb)

## Abbreviations

co-DEG: co-differentially expressed gene
TF: Transcription factors
GA: Gibberellin
JA: Jasmonic acid
ET: Ethylene
