# Supplementary material for: Transcriptome profile analysis of cell proliferation molecular processes during multicellular trichome formation induced by tomato Wov gene in tobacco
Source: BMC Genomics. 2015 Oct 26;16:868. doi: 10.1186/s12864-015-2099-7 (PMC4623907; doi:10.1186/s12864-015-2099-7)
Supplement: Additional file 1: — The sequence information of wo gene and its allele Wo v gene. (DOCX 17 kb) [file 12864_2015_2099_MOESM1_ESM.docx]

**> *Wo*^v^**

ATGTTTAATAACCACCAGCACTTGCTCGATATATCGTCCTCAGCTCAACGAACACCTGATAACGAGTTGGATTTCATTCGTGATGAAGAGTTTGATAGCAACTCTGGTGCTGATAACATGGAAGCTCCCAATTCAGGTGATGACGATCAAGCTGATCCAAACCAACCTCCAAACAAGAAGAAGCGTTATCATCGCCACACTCAGAATCAGATTCAGGAAATGGAGTCCTTTTACAAGGAATGCAATCATCCAGATGACAAGCAAAGGAAGGAATTGGGAAGAAGACTTGGTTTGGAGCCATTACAAGTGAAATTTTGGTTCCAGAACAAGCGTACTCAGATGAAGGCTCAACATGAGCGATGTGAGAACACACAGTTGAGGAATGAAAATGAGAAGCTTCGCGCTGAGAACATAAGGTACAAAGAAGCTTTGAGTAATGCAGCATGCCCAAATTGTGGAGGGCCAGCAGCTATAGGAGAGATGTCATTTGATGAGCATCAGTTGAGGATTGAAAATGCTCGTCTTAGAGATGAGATTGACAGGATAACTGGAATAGCTGGAAAGTATGTTGGTAAATCAGCCCTTGGATATTCTCATCAACTTCCTCTTCCTCAGCCCGAAGCTCCTCGGGTTCTGGATCTTGCTTTTGGGCCTCAATCGGGCCTGCTTGGAGAAATGTACGCTGCTGGTGACCTTCTAAGAACTGCTGTTACGGGCCTTACAGATGCTGAGAAGCCCGTGGTCATTGAGCTTGCTGTTACTGCAATGGAGGAACTTATAAGGATGGCTCAAACTGAAGAGCCATTATGGTTGCCAAGCTCAGGCTCTGAGACTTTATGTGAGCAAGAATATGCTCGTATTTTCCCTCGAGGCCTTGGACCTAAGCCAGCTACACTCAATTCTGAAGCCTCACGAGAATCTGCTGTTGTGATTATGAATCATATCAATTTAGTTGAGATTTTGATGGATGTGAACCAATGGACTACTGTTTTTGCTGGTCTGGTGTCAAAAGCAATGACTCTTGAAGTCTTATCAACTGGTGTCGCAGGAAATCACAATGGAGCATTGCAAGTGATGACAGCAGAATTTCAAGTTCCATCTCCACTTGTTCCAACTCGGGAGAACTATTTCTTAAGATACTGTAAACAACATGGTGAAGGGACTTGGGTAGTGGTTGATGTTTCCCTGGACAACTTGCGCACTGTTTCAGTTCCGCGTTGCAGAAGAAGGCCATCTGGTTGTTTAATCCAAGAAATGCCAAATGGTTACTCAAGGGTTATATGGGTTGAACACGTTGAGGTGGATGAAAATGCTGTCCATGACATCTACAAACCTCTTGTCAATTCTGGGATTGCATTTGGAGCAAAACGCTGGGTAGCAACTTTAGATAGACAATGTGAACGCCTTGCAAGTGTGTTGGCGCTTAACATCCCAACAGGAGATGTTGGAATCATTACTAGTCCAGCTGGTCGAAAGAGTATGCTAAAACTTGCTGAGAGAATGGTGATGAGCTTTTGTGCTGGAGTTGGTGCATCGACAACTCACATATGGACAACTTTGTCTGGAAGTGGTGCGGATGATGTTAGAGTCATGACTAGGAAGAGTATCGATGATCCAGGGAGACCTCCTGGTATTGTGCTGAGTGCTGCAACATCTTTTTGGCTTCCAGTTTCTCCTAAGAGAGTGTTTGATTTTCTCCGCGATGAGAACTCTAGAAATGAGTGGGATATTCTTTCAAATGGTGGGATTGTTCAGGAAATGGCACACATTGCAAATGGTCGTGATCCAGGAAACTGTGTTTCTCTACTCCGTGTCAATACTGGAACAAACTCTAACCAGAGTAACATGCTGATACTCCAAGAGAGCACAACTGATGTAACAGGATCTTACGTCATTTACGCTCCAGTTGATATTGCTGCAATGAACGTGGTGTTAGGTGGGGGTGACCCTGACTATGTTGCTCTGTTGCCATCTGGTTTTGCTATTCTTCCAGACGGACCGATGAATTATCATGGTGGAGGTAATTCAGAAATTGATTCTCCTGGTGGATCGCTACTAACTGTAGCATTTCAGA**G**ATTGGTT**T**ATTCAGTCCCAACTGCAAAGCTTTCCCTTGGCTCTGTTGCGACTGTTAATAGTCTCATCAAATGCACCGTTGAAAAGATCAAAGGTGCTGTAACTTCCGCAAATGCATGA

MFNNHQHLLDISSSAQRTPDNELDFIRDEEFDSNSGADNMEAPNSGDDDQADPNQPPNKKKRYHRHTQNQIQEMESFYKECNHPDDKQRKELGRRLGLEPLQVKFWFQNKRTQMKAQHERCENTQLRNENEKLRAENIRYKEALSNAACPNCGGPAAIGEMSFDEHQLRIENARLRDEIDRITGIAGKYVGKSALGYSHQLPLPQPEAPRVLDLAFGPQSGLLGEMYAAGDLLRTAVTGLTDAEKPVVIELAVTAMEELIRMAQTEEPLWLPSSGSETLCEQEYARIFPRGLGPKPATLNSEASRESAVVIMNHINLVEILMDVNQWTTVFAGLVSKAMTLEVLSTGVAGNHNGALQVMTAEFQVPSPLVPTRENYFLRYCKQHGEGTWVVVDVSLDNLRTVSVPRCRRRPSGCLIQEMPNGYSRVIWVEHVEVDENAVHDIYKPLVNSGIAFGAKRWVATLDRQCERLASVLALNIPTGDVGIITSPAGRKSMLKLAERMVMSFCAGVGASTTHIWTTLSGSGADDVRVMTRKSIDDPGRPPGIVLSAATSFWLPVSPKRVFDFLRDENSRNEWDILSNGGIVQEMAHIANGRDPGNCVSLLRVNTGTNSNQSNMLILQESTTDVTGSYVIYAPVDIAAMNVVLGGGDPDYVALLPSGFAILPDGPMNYHGGGNSEIDSPGGSLLTVAFQRLVYSVPTAKLSLGSVATVNSLIKCTVEKIKGAVTSANA

**>*wo***

ATGTTTAATAACCACCAGCACTTGCTCGATATATCGTCCTCAGCTCAACGAACACCTGATAACGAGTTGGATTTCATTCGTGATGAAGAGTTTGATAGCAACTCTGGTGCTGATAACATGGAAGCTCCCAATTCAGGTGATGACGATCAAGCTGATCCAAACCAACCTCCAAACAAGAAGAAGCGTTATCATCGCCACACTCAGAATCAGATTCAGGAAATGGAGTCCTTTTACAAGGAATGCAATCATCCAGATGACAAGCAAAGGAAGGAATTGGGAAGAAGACTTGGTTTGGAGCCATTACAAGTGAAATTTTGGTTCCAGAACAAGCGTACTCAGATGAAGGCTCAACATGAGCGATGTGAGAACACACAGTTGAGGAATGAAAATGAGAAGCTTCGCGCTGAGAACATAAGGTACAAAGAAGCTTTGAGTAATGCAGCATGCCCAAATTGTGGAGGGCCAGCAGCTATAGGAGAGATGTCATTTGATGAGCATCAGTTGAGGATTGAAAATGCTCGTCTTAGAGATGAGATTGACAGGATAACTGGAATAGCTGGAAAGTATGTTGGTAAATCAGCCCTTGGATATTCTCATCAACTTCCTCTTCCTCAGCCCGAAGCTCCTCGGGTTCTGGATCTTGCTTTTGGGCCTCAATCGGGCCTGCTTGGAGAAATGTACGCTGCTGGTGACCTTCTAAGAACTGCTGTTACGGGCCTTACAGATGCTGAGAAGCCCGTGGTCATTGAGCTTGCTGTTACTGCAATGGAGGAACTTATAAGGATGGCTCAAACTGAAGAGCCATTATGGTTGCCAAGCTCAGGCTCTGAGACTTTATGTGAGCAAGAATATGCTCGTATTTTCCCTCGAGGCCTTGGACCTAAGCCAGCTACACTCAATTCTGAAGCCTCACGAGAATCTGCTGTTGTGATTATGAATCATATCAATTTAGTTGAGATTTTGATGGATGTGAACCAATGGACTACTGTTTTTGCTGGTCTGGTGTCAAAAGCAATGACTCTTGAAGTCTTATCAACTGGTGTCGCAGGAAATCACAATGGAGCATTGCAAGTGATGACAGCAGAATTTCAAGTTCCATCTCCACTTGTTCCAACTCGGGAGAACTATTTCTTAAGATACTGTAAACAACATGGTGAAGGGACTTGGGTAGTGGTTGATGTTTCCCTGGACAACTTGCGCACTGTTTCAGTTCCGCGTTGCAGAAGAAGGCCATCTGGTTGTTTAATCCAAGAAATGCCAAATGGTTACTCAAGGGTTATATGGGTTGAACACGTTGAGGTGGATGAAAATGCTGTCCATGACATCTACAAACCTCTTGTCAATTCTGGGATTGCATTTGGAGCAAAACGCTGGGTAGCAACTTTAGATAGACAATGTGAACGCCTTGCAAGTGTGTTGGCGCTTAACATCCCAACAGGAGATGTTGGAATCATTACTAGTCCAGCTGGTCGAAAGAGTATGCTAAAACTTGCTGAGAGAATGGTGATGAGCTTTTGTGCTGGAGTTGGTGCATCGACAACTCACATATGGACAACTTTGTCTGGAAGTGGTGCGGATGATGTTAGAGTCATGACTAGGAAGAGTATCGATGATCCAGGGAGACCTCCTGGTATTGTGCTGAGTGCTGCAACATCTTTTTGGCTTCCAGTTTCTCCTAAGAGAGTGTTTGATTTTCTCCGCGATGAGAACTCTAGAAATGAGTGGGATATTCTTTCAAATGGTGGGATTGTTCAGGAAATGGCACACATTGCAAATGGTCGTGATCCAGGAAACTGTGTTTCTCTACTCCGTGTCAATACTGGAACAAACTCTAACCAGAGTAACATGCTGATACTCCAAGAGAGCACAACTGATGTAACAGGATCTTACGTCATTTACGCTCCAGTTGATATTGCTGCAATGAACGTGGTGTTAGGTGGGGGTGACCCTGACTATGTTGCTCTGTTGCCATCTGGTTTTGCTATTCTTCCAGACGGACCGATGAATTATCATGGTGGAGGTAATTCAGAAATTGATTCTCCTGGTGGATCGCTACTAACTGTAGCATTTCAGA**T**ATTGGTT**G**ATTCAGTCCCAACTGCAAAGCTTTCCCTTGGCTCTGTTGCGACTGTTAATAGTCTCATCAAATGCACCGTTGAAAAGATCAAAGGTGCTGTAACTTCCGCAAATGCATGA

MFNNHQHLLDISSSAQRTPDNELDFIRDEEFDSNSGADNMEAPNSGDDDQADPNQPPNKKKRYHRHTQNQIQEMESFYKECNHPDDKQRKELGRRLGLEPLQVKFWFQNKRTQMKAQHERCENTQLRNENEKLRAENIRYKEALSNAACPNCGGPAAIGEMSFDEHQLRIENARLRDEIDRITGIAGKYVGKSALGYSHQLPLPQPEAPRVLDLAFGPQSGLLGEMYAAGDLLRTAVTGLTDAEKPVVIELAVTAMEELIRMAQTEEPLWLPSSGSETLCEQEYARIFPRGLGPKPATLNSEASRESAVVIMNHINLVEILMDVNQWTTVFAGLVSKAMTLEVLSTGVAGNHNGALQVMTAEFQVPSPLVPTRENYFLRYCKQHGEGTWVVVDVSLDNLRTVSVPRCRRRPSGCLIQEMPNGYSRVIWVEHVEVDENAVHDIYKPLVNSGIAFGAKRWVATLDRQCERLASVLALNIPTGDVGIITSPAGRKSMLKLAERMVMSFCAGVGASTTHIWTTLSGSGADDVRVMTRKSIDDPGRPPGIVLSAATSFWLPVSPKRVFDFLRDENSRNEWDILSNGGIVQEMAHIANGRDPGNCVSLLRVNTGTNSNQSNMLILQESTTDVTGSYVIYAPVDIAAMNVVLGGGDPDYVALLPSGFAILPDGPMNYHGGGNSEIDSPGGSLLTVAFQILVDSVPTAKLSLGSVATVNSLIKCTVEKIKGAVTSANA
