# Supplementary figures and images for: Transcriptome profile analysis of cell proliferation molecular processes during multicellular trichome formation induced by tomato Wov gene in tobacco
Source: BMC Genomics. 2015 Oct 26;16:868. doi: 10.1186/s12864-015-2099-7 (PMC4623907; doi:10.1186/s12864-015-2099-7)

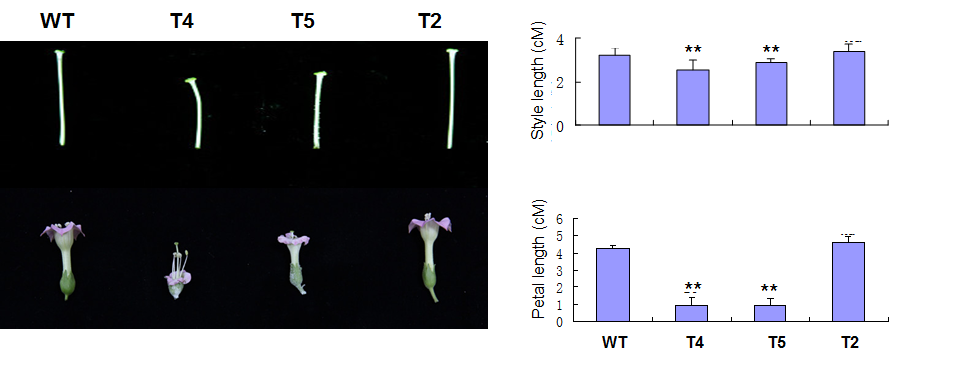

Supplement: Additional file 3: — Wo v ectopic expression in tobacco plants (T4, T5 and T2) causes the formation of malformed flower, such as shorter style and shorter petal than wild type (WT). (TIFF 104 kb) [file 12864_2015_2099_MOESM3_ESM.tif]

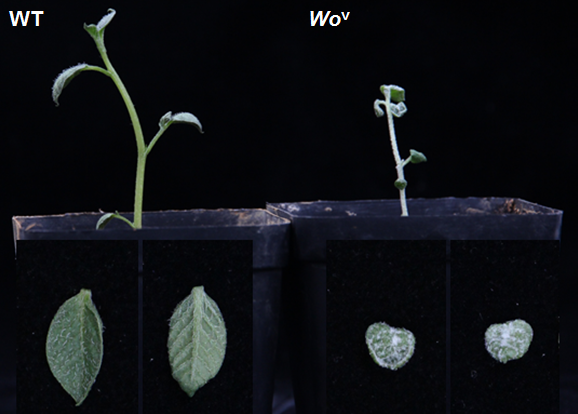

Supplement: Additional file 4: — Trichome phenotype of Wo v ectopically expressed potato ( Wo v ) and wild type plants (WT). (TIFF 278 kb) [file 12864_2015_2099_MOESM4_ESM.tif]

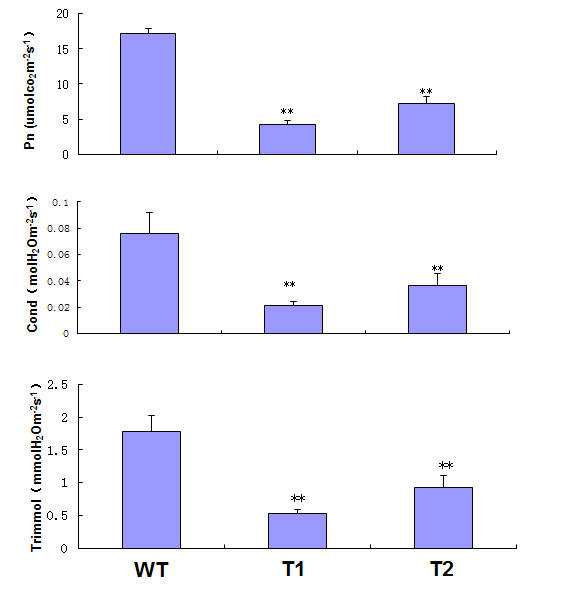

Supplement: Additional file 5: — Comparison of the rate of photosynthesis, stomatal conductance and transpiration rate between Wo v transgenic tobacco plants (T1 and T2) and wild type (WT). (TIFF 12 kb) [file 12864_2015_2099_MOESM5_ESM.tif]

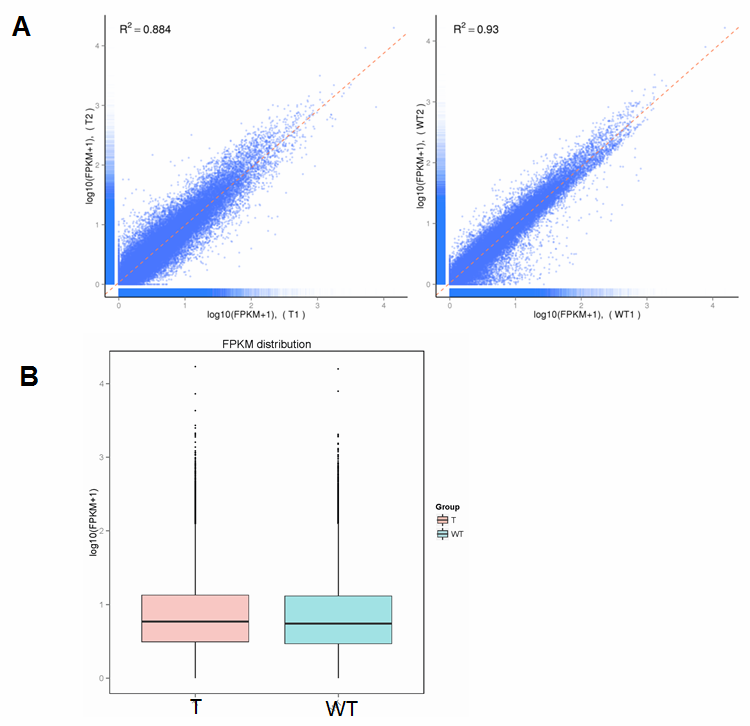

Supplement: Additional file 6: — Analysis of the gene expression correlation between the two indicated pairs of samples (T1 versus WT1, T2 versus WT2) (A), and boxplot of the log transformed FPKM expression values across four samples (B). (TIFF 100 kb) [file 12864_2015_2099_MOESM6_ESM.tif]

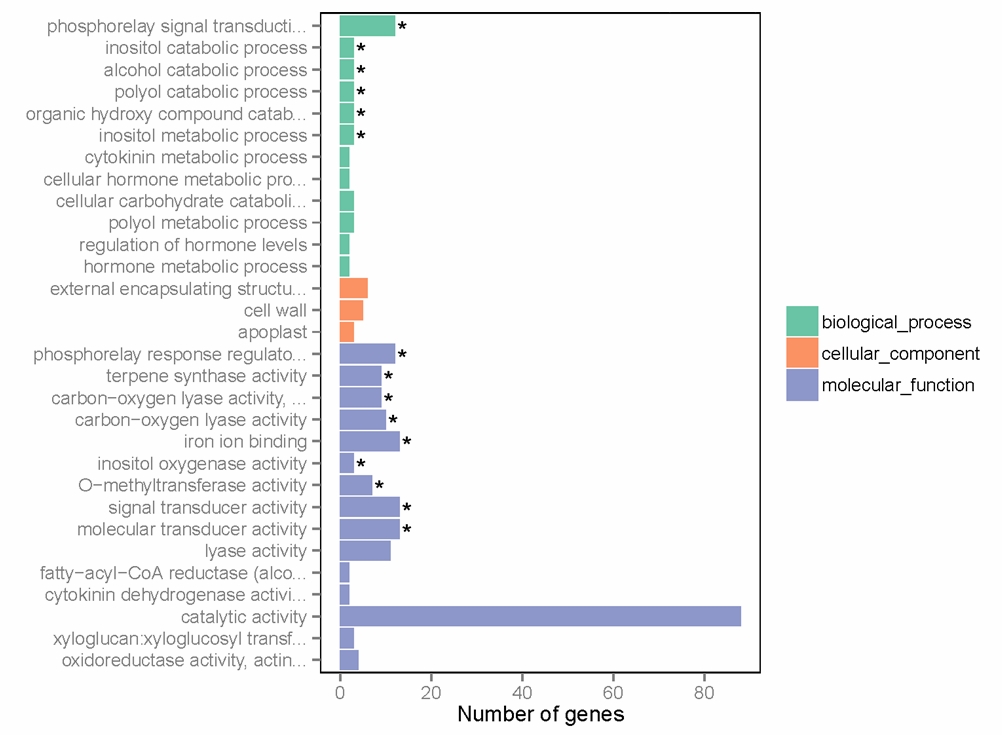

Supplement: Additional file 8: — Functional categorization of the down-regulated genes between the Wo v transgenic tobacco plants and the wild-type. (JPEG 203 kb) [file 12864_2015_2099_MOESM8_ESM.jpg]
